# Case Report: Biallelic *PADI6* frameshift variants contribute to preimplantation embryonic lethality

**DOI:** 10.3389/fgene.2026.1667814

**Published:** 2026-03-11

**Authors:** Shun-Tao Jiao, Zi-Hui Tan, Tian-Ying Wei, Ge-Han Zhang, Ti-Ling Hu, Song-Jun Li, Ya-Ping Tian, Jia-En Liu, Hua-Ying Hu

**Affiliations:** 1 Beijing Jiaen Hospital, Heen Life Medical Research Institute, Beijing, China; 2 Medical Innovation Research Division of Chinese PLA General Hospital, Beijing, China; 3 The Reproduction Medical Center, The Third Affiliated Hospital of Shenzhen University, Shenzhen, Guangdong, China

**Keywords:** molecular dynamics (MD), PADI6, preimplantation embryonic lethality, protein function analysis, whole-exome sequencing (WES)

## Abstract

**Objectives:**

Preimplantation embryonic lethality (PREMBL) is a major cause of female infertility, characterized by early embryonic arrest. Homozygous or compound heterozygous mutations in *PADI6* underlie preimplantation embryonic lethality-2 (PREMBL2). This study aims to conduct a systematic genetic investigation on a case with *PADI6* biallelic variants.

**Methods:**

A patient clinically diagnosed with PREMBL was systematically evaluated via chromosomal karyotyping analysis, high-resolution chromosomal microarray analysis (CMA), whole-exome sequencing (WES), molecular dynamics analysis (MD), immunofluorescence (IF), and Western blotting (WB) to identify PREMBL-associated variants.

**Results:**

WES identified two biallelic frameshift variants in *PADI6*: c.707dupT (L237Afs*24) and c.2009_2010delAG (E670Gfs*48). MD, IF, and WB analyses demonstrated that: The L237A variant generates a 259-aa truncated protein lacking the essential C-terminal domain. The E670G variant produces a 716-aa elongated protein with significant C-terminal structural alterations. Both variants cause complete loss of protein function and markedly reduced abundance.

**Conclusion:**

This study implicates a previously unreported compound heterozygous combination of the *PADI6* variants c.707dupT and c.2009_2010delAG as the potential genetic basis of PREMBL2 in this individual, based on their predicted disruptive effects. However, as a single-case study, these findings cannot be generalized. Future research should focus on validating these variants in additional patients and functionally characterizing their effects to definitively establish causality and understand their role in early embryonic arrest.

## Introduction

The normal development of the preimplantation embryo is a critical step for achieving a successful pregnancy. Early embryonic arrest is a major contributor to female infertility. Preimplantation Embryonic Lethality (PREMBL), also termed early embryonic arrest, constitutes a significant cause of female infertility (Xu et al., 2016). However, pinpointing the genes responsible for human embryonic lethality is challenging, particularly when the associated phenotype manifests during the preimplantation stage (Alazami et al., 2015). The advancement of assisted reproductive technology (ART) now enables detailed assessment of the phenotypic characteristics associated with early human embryonic arrest.

Preimplantation embryonic lethality-1 (PREMBL1, MIM #616814) is caused by homozygous mutations in the *TLE6* gene (MIM *612399) located on chromosome 19p13. *TLE6* gene mutations represent a rare cause of human female infertility and constitute the earliest-appearing form of human embryonic lethality currently known to be caused by single-gene mutations. In affected women, ovulation occurs normally and the retrieved oocytes exhibit normal morphology; however, zygote formation is severely compromised (Alazami et al., 2015). Preimplantation embryonic lethality-2 (PREMBL2, MIM #617234) is caused by homozygous or compound heterozygous mutations in the *PADI6* gene (MIM *610363) located on chromosome 1p36. In 2016, Xu et al. reported the identification of homozygous or compound heterozygous truncating or missense mutations in the *PADI6* gene, found during screening of five infertile Chinese women (from three unrelated families) experiencing early embryonic arrest (Xu et al., 2016). Subsequently, *PADI6* gene mutations have been reported in additional PREMBL2 patients by several independent research groups (Zheng et al., 2020; Maddirevula et al., 2017; Qian et al., 2018; Wang et al., 2018).

PADI6 is a protein acting as a post-translational modification enzyme. In the presence of calcium ions, it converts arginine residues into citrulline residues (Chavanas et al., 2004). In mouse models, *PADI6* gene expression peaks during the 2-cell stage of embryonic development, coinciding with embryonic genome activation. Female mice with a homozygous deletion of the *PADI6* gene (*PADI6* ^−/−^) exhibit infertility due to early embryonic arrest (Yurttas et al., 2008; Kan et al., 2011). In humans, PADI6 protein is highly expressed in oocytes but shows only weak or undetectable expression in other somatic tissues and spermatozoa (Xu et al., 2016).

In this study, we recruited a couple affected by Preimplantation Embryonic Lethality (PREMBL) and performed standard genetic screening. This analysis identified biallelic variants within the *PADI6* gene. To confirm the pathogenicity of the identified variants, we conducted experiments including molecular dynamics analysis (MD), immunofluorescence (IF), quantitative fluorescent PCR (QF-PCR), and Western blotting (WB). These assays also clarified the impact of the variants on PADI6 protein structure and function.

## Materials and methods

### Subjects and clinical evaluation

This study (Approval No.: BJH-2024004) was approved by the Ethics Committee of Beijing Jiaen Hospital. All participants provided informed consent, with procedures complying with the Declaration of Helsinki (1964) and amendments.

In February 2024, a couple with PREMBL was enrolled at Beijing Jiaen Hospital. Family history was documented and the couple completed clinical examinations, laboratory tests, and basal hormone analysis (Cobas® 6000, Roche Diagnostics).

### Genetic analysis

Karyotyping (550-band G-banding), chromosomal microarray analysis (CMA, Cytoscan 750k), and whole-exome sequencing (WES, Illumina NovaSeq 6000) were performed on the couple.

Whole-exome sequencing (WES) was performed on the proband’s DNA sample to detect sequence variants, following a methodology previously described (Yang et al., 2019). Target regions were enriched using the Agilent Sure Select Human Exon Sequence Capture Kit (Agilent Technologies, United States). Enrichment efficiency of the DNA library was assessed by quantitative PCR. Library fragment size, distribution, and concentration were determined using the Agilent Bioanalyzer 2100 (Agilent Technologies, United States). Sequencing was performed on the NovaSeq6000 (Illumina, Inc.) platform with paired-end reads of approximately 150 bp. Libraries (normalized to ∼300 pM per sample) were sequenced using the NovaSeq Reagent kit. Raw sequencing reads (quality level Q30% > 90%) were aligned to the human reference genome (accession no: hg19/GRCh37) using the Burrows-Wheeler Aligner (BWA). PCR duplicates were marked and removed using Picard tools (v1.57). Variant calling was performed using the Verita Trekker® Variant Detection System (v2.0; Berry Genomics, China) in conjunction with the Genome Analysis Tool Kit (https://software.broadinstitute.org/gatk/). Variant annotation and interpretation were conducted following the joint guidelines of the American College of Medical Genetics and Genomics (ACMG) (Richards et al., 2015), utilizing ANNOVAR v2.0 (Wang et al., 2010) and the Enliven® Variant Annotation Interpretation System (Berry Genomics). Variant pathogenicity assessment was supplemented using data from the three frequency databases (1000G_2015aug_eas, https://www.internationalgenome.org; ExAC_EAS, http://exac.broadinstitute.org; gnomAD_exome_EAS, http://gnomad.broadinstitute.org), as well as the Human Gene Mutation Database (HGMD) pro v2019. Additionally, the Revel score (for pathogenicity prediction) (Ioannidis et al., 2016) and the pLI score (representing the tolerance for truncating variants) were applied.

Sanger sequencing, performed on an Applied Biosystems 3500DX Genetic Analyzer (Thermo Fisher Scientific, United States), was used to validate the identified variants.

### *In silico* protein structural and molecular dynamic analysis

The impact of missense variants on amino acid evolutionary conservation was analyzed using MEGA7 software (http://www.megasoftware.net/previousVersions.php; default parameters). The conservation score of specific amino acid residue positions was visualized using the WEBLOGO tool (http://weblogo.berkeley.edu/logo.cgi).

Homology modeling was performed using SWISS-MODEL, based on the crystal structure template PDB: AF-Q6TGC4-F1 (https://swissmodel.expasy.org), to generate three-dimensional (3D) structural models of the wild-type (WT) PADI6 protein and the mutant proteins (p.L237Afs*24 and p.E670Gfs*48).

Subsequently, molecular dynamics analysis (MD) were conducted on the wild-type (WT) and mutant PADI6 models (p.L237Afs*24 and p.E670Gfs*48) generated by Modeller 9v17 (Sali et al., 1995). Hydrogen atoms and N-/C-terminal patches were added using the CHARMM22 force field (MacKerell et al., 1998). The models were solvated in a TIP3P water box for hydration and charge neutralization, with the minimum distance between the model and the box boundary set to 13 Å. Simulations were performed using the NAMD 2.9 software under periodic boundary conditions (PBC) (Phillips et al., 2020). Key simulation parameters included: temperature maintained at 300 K, pressure at 1 atmosphere (atm), and time steps of 2 femtoseconds (fs). Electrostatic interactions were modeled using the particle mesh Ewald (PME) method, with a van der Waals interaction cutoff distance of 12 Å. The models underwent a three-stage pre-equilibration process (total duration: 600 ps), the last snapshots were selected as the beginning structures for 40 ns production simulations under unrestrained conditions.

### Plasmids construction and cell transfection

To construct expression plasmids encoding wild-type (WT) or mutant PADI6 (p.L237Afs*24 and p.E670Gfs*48), total RNA was extracted from the proband’s mRNA sample. WT and mutant PADI6 cDNA sequences were amplified by RT-PCR. The resulting cDNA fragments were subcloned into the pET vector. After verification by Sanger sequencing, the fragments were cloned into the pcDNA3.1(+)-3×Flag vector. The final constructs were designated as PADI6-WT, PADI6-L237Afs*24 and PADI6-E670Gfs*48.

HEK 293T cells (ATCC, Cat# CRL-3216) were cultured and seeded in 24-well plates. Upon reaching appropriate confluency, cells were transfected with the respective plasmids using Lipofectamine 3000(Invitrogen, United States). Cells were harvested at 36 or 48 h post-transfection for subsequent analysis.

### Quantitative fluorescent PCR analysis

Cells were harvested 48 h post-transfection. Total RNA was extracted using the RNeasy Mini Kit (QIAGEN), followed by cDNA synthesis with the PrimeScript RT Reagent Kit (including gDNA Eraser, Takara). Gene expression levels were quantified using gene-specific primers, normalized to the reference gene GAPDH (MIM:138400). Real-time qPCR was performed on a 7500 Fast Real-Time PCR System (Applied Biosystems) with three technical replicates per sample. Data are presented as mean ± standard deviation (SD) from three independent experiments. The PCR cycle was as follows: 95 °C for 10 min, 1 cycle; 95 °C for 10 s + 60 °C for 30 s (fluorescence acquisition), 55 cycles. The expression values of each gene were normalized to the expression level of GAPDH using the 2^−ΔΔCT^ method.

### Immunofluorescence analysis

At 36 h post-transfection, cells were fixed with 4% paraformaldehyde for 30 min, followed by permeabilization with 1% Triton X-100 at room temperature for 1 h. Samples were blocked in blocking buffer (3% BSA and 5% goat serum in PBS) for 1 h. Primary incubation was performed with anti-Flag mouse monoclonal antibody (1:400 dilution; Sigma-Aldrich, United States) at 4 °C overnight. After PBS washes, samples were incubated with Alexa Fluor-conjugated goat anti-mouse secondary antibody (1:400 dilution; Invitrogen, Carlsbad, CA) for 2 h at room temperature protected from light. Nuclei were counterstained with DAPI (Invitrogen, Carlsbad, CA) for 5 min. Coverslips were mounted using Fluoromount aqueous mounting medium (Sigma-Aldrich, St Louis, WA) and imaged under a laser scanning confocal microscope (Zeiss, Gottingen, Germany) with a ×63 oil immersion objective.

### Western blotting analysis

Cells were harvested 48 h post-transfection and lysed with 2× SDS lysis buffer containing 1 mM phenylmethanesulfonyl fluoride (PMSF) (Sigma, St Louis, WA), 0.2 mM β-mercaptoethanol, and protease inhibitor cocktail (Sigma, St Louis, WA). Equal protein lysates were resolved by 10% sodium dodecyl sulfate-polyacrylamide gel electrophoresis (SDS-PAGE), transferred onto polyvinylidene fluoride (PVDF) membranes, and subjected to immunoblotting with anti-Flag mouse monoclonal antibody. β-actin served as the loading control.

### Statistical analysis

Statistical analyses were performed using Prism 8 software (GraphPad Software, San Diego, CA). Intergroup differences were evaluated by Student's *t*-tests. Statistical significance was defined as *p* ≤ 0.05.

## Results

### Clinical presentation

The pedigree chart constructed through systematic collection of family history is presented in Figure 3A. The proband (II-1, female) and her spouse (II-2, male) were both 33 years old with a 5-year history of infertility. Body mass index (BMI) and basal hormone levels were within normal ranges for both individuals. Gynecological examination revealed right unilateral hydrosalpinx in II-1. For II-2, no erectile or ejaculatory dysfunction was reported, and semen kinetic analysis demonstrated normal sperm motility and morphological parameters. Both individuals denied a history of sexually transmitted infections (STIs), long-term medication use, or exposure to harmful chemicals or radiation.

**FIGURE 1:**
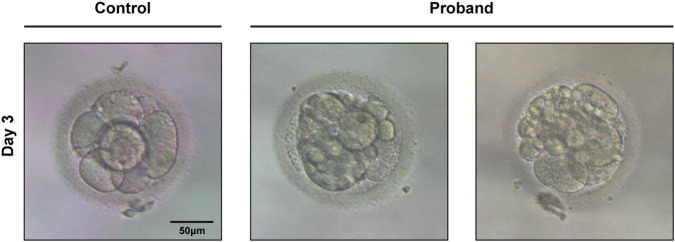
Embryo from the healthy female (control group) and embryos with early developmental arrest (proband group) on day 3 post-*in vitro* fertilization.

The couple underwent two *in vitro* fertilization (IVF) cycles, both of which employed a GnRH antagonist protocol. In the first cycle, twelve oocytes were retrieved, eight of which were successfully fertilized and progressed to cleavage. The second cycle yielded fifteen oocytes, with nine achieving successful fertilization and undergoing cleavage. Crucially, in both cycles, all embryos that successfully fertilized and reached the cleavage stage exhibited developmental arrest on culture day 3 (at the 4 to 8-cell stage) (Figure 1).

### Cytogenetic and molecular genetic findings

**FIGURE 2:**
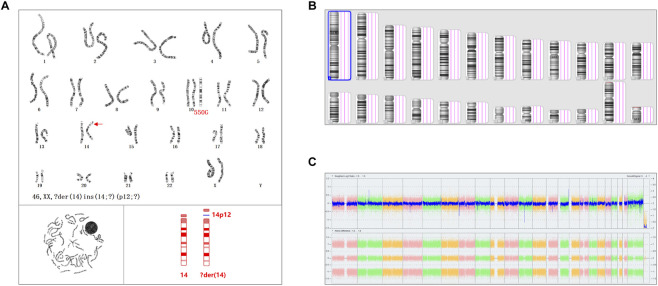
Results of karyotype and chromosomal microarray analysis (CMA). **(A)** Karyotype results. The arrow indicates the suspected insertion of an unknown fragment in chromosome 14; 550G represents the resolution of the karyotype. **(B)** Virtual chromosomal karyotype by CMA. **(C)** Whole-genome signal intensity-weighted ratio by CMA.

Chromosomal analysis at 550-band resolution revealed a normal male karyotype (46,XY) (Figure 2A). The female karyotype was reported as 46,XX,?der(14)ins(14;?)(p12;?), suggesting an insertion of unknown origin at chromosome 14p12. Subsequent high-resolution chromosomal microarray analysis (CMA) confirmed a normal female genomic copy number profile arr(1-22,X)×2, leading to the classification of this karyotypic abnormality as a clinically insignificant benign polymorphism (Figures 2B,C).

**FIGURE 3:**
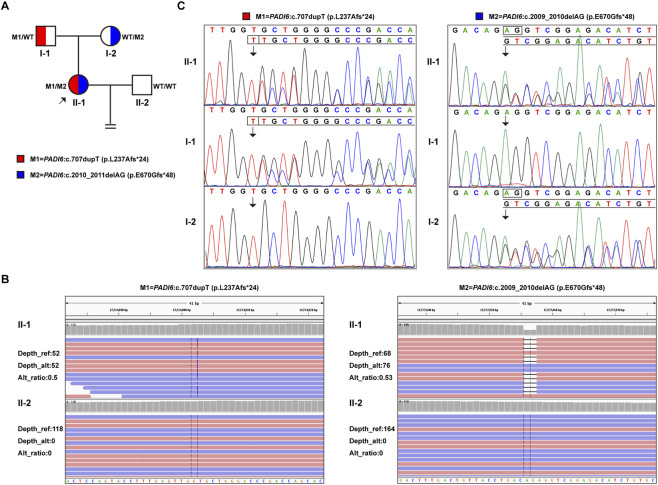
WES outcomes and Sanger sequencing validation results. **(A)** Pedigree of the case. The arrow denotes the proband (II-1) with compound heterozygous variants, including maternally inherited c.2009_2010delAG (E670Gfs*48) and paternally inherited c.707dupT (L237Afs*24). **(B)** WES results of the proband (II-1) and his spouse (II-2). **(C)** Sanger sequencing validation results of the maternal variant c.2009_2010delAG (E670Gfs*48) and the paternal variant c.707dupT (L237Afs*24).

WES identified no pathogenic variants in the male partner (Figure 3B). In the female proband, compound heterozygous frameshift variants in the *PADI6* gene (RefSeq transcript NM_207421.4) were detected: c.707dupT (L237Afs*24) and c.2009_2010delAG (E670Gfs*48). Both variants induce a frameshift. The c.707dupT variant, inherited from the proband’s father, results in a premature termination codon (PTC) and predicted protein truncation (Figures 3A,I–1). Conversely, the c.2009_2010delAG variant, inherited from the proband’s mother, leads to a delayed termination codon and predicted protein elongation (Figures 3A,I–2). Following the American College of Medical Genetics and Genomics (ACMG) guidelines, these variants were classified as “Likely Pathogenic” (PVS1+PM2+PP4) and “Pathogenic” (PVS1+PM2+PP4), respectively. This classification was based on their loss-of-function mechanism, absence in population databases (gnomAD), and their phenotypic correlation with Preimplantation Embryonic Lethality 2 (PREMBL2; OMIM: #616814), an autosomal recessive disorder. Database searches confirmed that this specific compound heterozygous combination has not been previously reported.

### Conservation analysis and structural differences of PADI6 L237A and E670G variants

**FIGURE 4:**
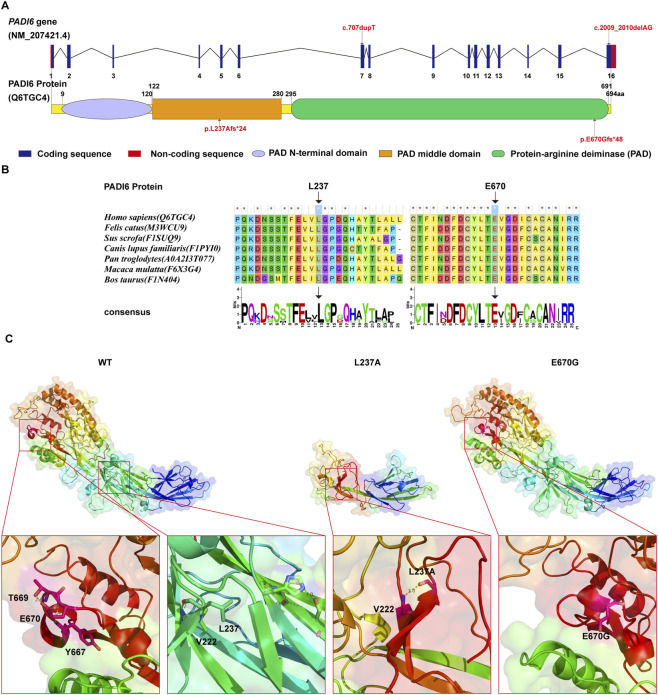
Schematic of *PADI6* gene and its encoded protein, conservation analysis results, and the simulated three-dimensional structure of PADI6. **(A)** Schematic of the *PADI6* gene and its encoded protein. The arrows mark the positions of the mutation sites in this case. **(B)** Multiple sequence alignment of PADI6 residues across seven species using MEGA7 software. **(C)** Three-dimensional models of wild-type (WT) PADI6 protein and mutants p.L237Afs*24 (L237A)/p.E670Gfs*48 (E670G) constructed using SWISS-MODEL. The stick model shows L237, E670, mutated residues, and interacting residues. The yellow dotted lines indicate hydrogen bonds involving the variant sites and the corresponding WT amino acids.

Multiple sequence alignment of PADI6 protein sequences from seven species (Figure 4B). The results demonstrated that Leu237 (L237) and Glu670 (E670) exhibited 100% conservation scores across all analyzed species. Visualization of the ±12 amino acid regions flanking these residues using WEBLOGO revealed single dominant peak characteristics for both positions, indicating strong purifying selection during evolution.

Homology modeling via the SWISS-MODEL server was employed to generate three-dimensional structures of wild-type (WT) PADI6 and its variants p.L237Afs*24 (L237A) and p.E670Gfs*48 (E670G) (Figure 4C). The p.L237Afs*24 mutation localizes to the intermediate domain of PADI6, where the introduction of a premature termination codon results in a truncated protein backbone of 259 amino acids. This represents a 62.7% reduction in length compared to the 694-amino-acid WT protein and leads to complete ablation of the C-terminal domain (Figure 4A).

The p.E670Gfs*48 variant resides within the C-terminal domain (Figure 4A). This mutation extends the protein backbone to 716 amino acids, incorporating 22 additional residues at the C-terminus relative to WT. Structural analysis reveals the disruption of two critical hydrogen bonds maintained between Glu670 and other residues in the wild-type (WT) conformation. Consequently, this alteration induces localized structural differences at the C-terminal region.

### Molecular dynamics study of PADI6 L237A and E670G variants

**FIGURE 5:**
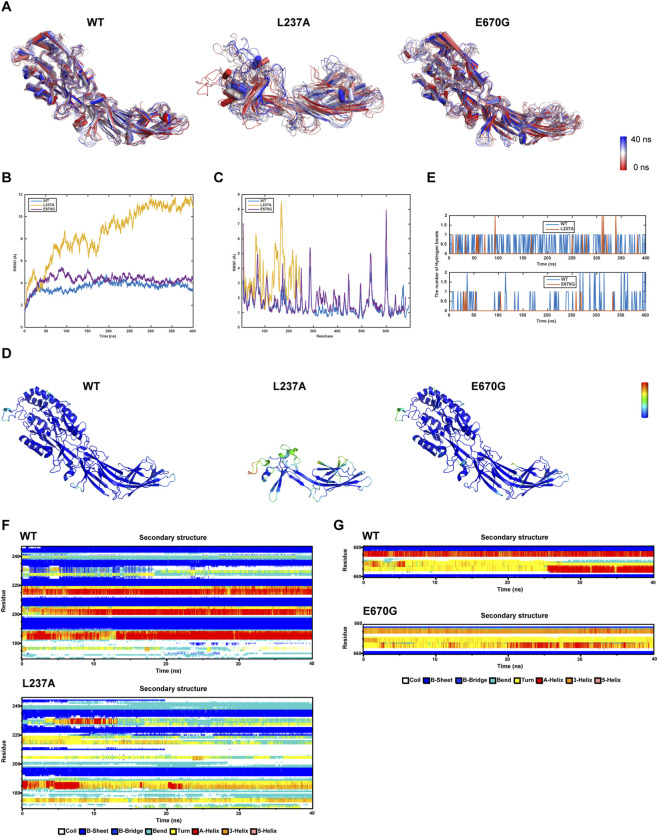
Molecular dynamics analysis (MD) of wild-type (WT) PADI6 and mutants L237A/E670G. **(A)** Superimposed trajectory snapshots at 10 time points during 0–40 ns simulations. **(B)** Root mean square deviation (RMSD) of Cα atoms over 400 ns simulations. **(C,D)** Root mean square fluctuation (RMSF) profiles and analytical results. **(E)** Hydrogen bond counts between mutated residues and neighboring residues. **(F,G)** Secondary structure transitions of key functional residues in WT and mutants during 0–40 ns simulations.

Molecular dynamics analysis (MD) during 0–40 ns revealed that the L237A mutant exhibited unstable overall conformations (Figure 5A). The E670G variant maintained relatively stable global conformations but developed localized structural changes at the C-terminus during later simulation stages, which was corroborated by root mean square deviation (RMSD) trajectories and root mean square fluctuation (RMSF) analysis (Figures 5B–D).

Further RMSF analysis indicated enhanced flexibility of key residues and structural instability in critical local regions of L237A, specifically manifested by near-complete α-helix disappearance, reduced β-sheet content, and increased random coils/turns beyond residue 170 (Figure 5F). While E670G showed dynamic distributions similar to WT overall, it exhibited higher residue flexibility in certain regions. Pronounced structural fluctuations emerged beyond residue 660, characterized by destabilized α-helices and diminished β-sheets (Figure 5G).

Both variants demonstrated reduced hydrogen bonding between mutation sites and neighboring residues compared to WT (Figure 5E). In summary, L237A and E670G affect protein structural integrity and stability to varying degrees.

### Protein expression and molecular weight studies of PADI6 L237A and E670G variants

**FIGURE 6:**
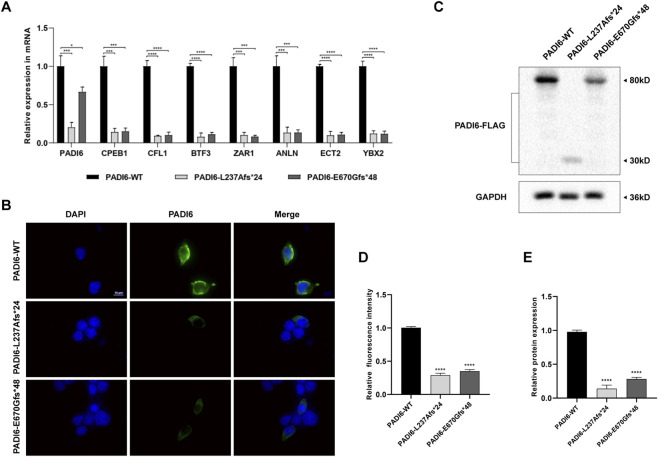
Functional analysis of mutant proteins. **(A)** Relative mRNA expression levels of *PADI6* wild-type (WT), mutants p.L237Afs*24 (L237A)/p.E670Gfs*48 (E670G), and *PADI6*-interacting genes. **(B)** HEK293 cells transiently transfected with pcDNA3.1-PADI6-WT/L237A/E670G plasmids (Scale bar = 10 μm). **(C)** Immunoblot analysis of transiently expressed PADI6-WT/L237A/E670G proteins in HEK293 cells using anti-FLAG and anti-GAPDH antibodies. **(D)** Quantified relative fluorescence intensity of WT and mutant groups. **(E)** Relative PADI6 protein expression in WT and mutant groups (biological triplicates). Data analyzed by one-way ANOVA; *p < 0.05, **p < 0.01, ***p < 0.001, ****p < 0.0001; error bars denote mean ± SD.

Statistical analysis of mRNA expression levels in the wild-type group (PADI6-WT), L237A mutant group (PADI6-L237Afs*24), and E670G mutant group (PADI6-E670Gfs*48) revealed a reduction in relative mRNA expression for both mutants, with concomitant significant impairment in the expression of mRNAs encoding PADI6-interacting proteins (Figure 6A).

Subsequent immunofluorescence experiments demonstrated that neither the L237A nor E670G variants altered the subcellular localization of the protein, with both mutants exhibiting cytoplasmic distribution identical to WT (Figure 6B). However, comparative analysis of relative fluorescence intensity revealed significantly attenuated fluorescence signals in both mutants (Figure 6D).

Further Western blot analysis detected bands for both WT and E670G mutants at approximately 80 kDa, whereas the L237A mutant band at around 30 kDa, consistent with pre-experimental molecular weight expectations (WT: 80 kDa; L237A: 31 kDa; E670G: 82 kDa) (Figure 6C). Concurrently, the band signal intensities across three groups corroborated the immunofluorescence results. Statistical quantification of relative protein expression levels further confirmed significantly diminished expression in both mutants (Figure 6E).

Collectively, based on the results of protein expression, molecular weight, abundance and interacting proteins, it was confirmed that both mutations affect PADI6 function.

## Discussion

Previous studies have established that PADI6 and the subcortical maternal complex (SCMC)—composed of maternally encoded proteins including NLRP5, TLE6, OOEP and KHDC3 are critical for forming cytoplasmic lattices (Li et al., 2008; Esposito et al., 2007). These filamentous structures function not only to store proteins essential for embryonic development but also to sequester epigenetic regulators (e.g., UHRF1, KDM2B), ribosomal subunits, and metabolic enzymes. This sequestration prevents premature degradation or activation of stored proteins, ensuring their availability during the maternal-to-zygotic transition (MZT) and thereby supporting proper embryonic development (Jentoft et al., 2023).

In the present case, we identified compound heterozygous frameshift variants at two highly conserved sites, c.707dupT (L237Afs*24) and c.2009_2010delAG (E670Gfs*48), in a patient with a 5-year infertility history and recurrent embryonic developmental arrest at Day 3 (4 to 8-cell stage) across two failed *in vitro* fertilization (IVF) cycles. Through 3D structural modeling and molecular dynamics analysis (MD), we indicated that the c.707dupT variant introduces a premature stop codon, dramatically altering the overall protein architecture. While the c.2009_2010delAG variant delays termination codon formation, causing localized structural alteration near the C-terminus.

Immunofluorescence and Western blot showed that both variants disrupt PADI6 protein expression and its interactions. This aligns with qPCR data revealing lower mRNA levels for the two *PADI6* mutants and their interacting partners (Figure 6A). For *PADI6* itself, the c.707dupT variant likely triggers nonsense-mediated decay (NMD) due to its early premature stop codon. The c.2009_2010delAG variant, with a stop codon near the natural termination site, may escape strong NMD but still show reduced mRNA—possibly due to feedback regulation or mRNA instability. The decreased mRNA of interacting proteins likely reflects secondary effects of subcortical maternal complex (SCMC) destabilization, such as loss of mRNA stabilization (e.g., via disrupted PADI6-MSY2 interaction) or cellular feedback to maintain complex balance. We designed qPCR primers away from mutation sites, and the consistent downregulation across multiple SCMC-related genes supports a biological effect rather than technical artifact. Direct validation (e.g., mRNA stability assays) was not feasible in our human oocyte model. Therefore, these interpretations remain hypothetical and require future testing. Still, the combined protein and mRNA evidence provides a coherent molecular context for the pathogenic model below.

Based on these observations and existing literature, a potential pathogenic model can be proposed: the loss of PADI6 function may prevent proper higher-order assembly of the SCMC, potentially destabilizing the complex and leading to its diffuse cytoplasmic distribution. This, in turn, could compromise its incorporation into cytoplasmic lattices and disrupt the lattice structure (Jentoft et al., 2023). Such disruptions are hypothesized to deplete maternal protein stores, contribute to MZT failure, and ultimately manifest as arrested embryonic development before blastulation or other adverse reproductive outcomes (Bebbere et al., 2021; Seckl et al., 2010). In this specific case, the identified *PADI6* variants are consistent with the observed embryonic arrest at the 4–8 cell stage.

However, as a study based on a single case, it is crucial to interpret these findings with caution. While our analyses are consistent with a model where the identified *PADI6* variants contribute to the patient’s condition by potentially driving structural disruption and functional loss of PADI6, the evidence remains preliminary. The inherent limitations of a single-case study must be acknowledged. Specifically, the findings from this individual patient, while compelling, are insufficient to establish definitive genotype-phenotype correlations or broadly generalize the pathogenicity of these specific *PADI6* variants for PREMBL2. A causal link, while strongly suggested by the molecular evidence, requires definitive validation through functional studies in models and replication in larger patient cohorts.

Notwithstanding these limitations, this case provides valuable diagnostic clues for the genetic counseling of this family and underscores the potential clinical utility of an integrated cytogenetic and molecular diagnostic approach for investigating early embryonic arrest. It contributes a specific hypothesis regarding the molecular pathogenesis associated with these *PADI6* variants. Future studies prioritizing functional validation and investigation in larger populations are essential to confirm these observations and further elucidate the broader mechanisms of *PADI6*-related reproductive disorders.

## Conclusion

This study presents a patient with PREMBL2 in whom the compound biallelic *PADI6* mutations c.707dupT (L237Afs*24) and c.2009_2010delAG (E670Gfs*48) were identified. Comprehensive molecular analyses, including chromosomal karyotyping, CMA and WES provided evidence consistent with a model in which these variants may drive structural disruption and functional loss of *PADI6*. Although both variants have been individually reported, their compound heterozygous combination in a patient with PREMBL2 has not been previously described, highlighting the importance of evaluating compound heterozygosity in genetic diagnosis for infertility. While this finding offers valuable diagnostic evidence for the genetic counseling of this individual family and illustrates the potential utility of an integrated cytogenetic and molecular diagnostic framework for cases of early embryonic arrest, it is critical to emphasize the limitations inherent in a single-case study. The evidence, while compelling for this patient, is insufficient to establish definitive genotype-phenotype correlations or broadly generalize the pathogenicity of these *PADI6* variants for PREMBL2. Further validation in larger cohorts is essential to confirm these observations and explore the broader mechanisms of *PADI6*-related reproductive disorders.

## Data Availability

The datasets presented in this study can be found in online repositories. The names of the repository/repositories and accession number(s) can be found in the article/supplementary material.
